# Special Issue: Perovskite Nanostructures: From Material Design to Applications

**DOI:** 10.3390/nano12010097

**Published:** 2021-12-29

**Authors:** Athanasia Kostopoulou, Dimitra Vernardou

**Affiliations:** 1 Institute of Electronic Structure and Laser, Foundation for Research and Technology-Hellas, 71110 Heraklion, Greece; 2 Department of Electrical and Computer Engineering, School of Engineering, Hellenic Mediterranean University, 71004 Heraklion, Greece; 3 Institute of Emerging Technologies, Hellenic Mediterranean University Center, 71410 Heraklion, Greece

**Keywords:** nanocrystal growth techniques, characterization of perovskite structures, photoluminescence, metal-ion batteries, photovoltaics, organic solar cells

## Abstract

In the past decade, perovskite materials have attracted great scientific and technological interest due to their interesting opto-electronic properties. Nanostructuring of the perovskites, due to their reduced dimensions are advantageous in offering large surface area, controlled transport and charge carrier mobility, strong absorption and photoluminescence, and confinement effects. These features, together with the unique tunability in composition, shape, and functionalities in addition to the ability to form efficient, low-cost, and light-active structures make the perovskite nanostructures efficient functional components for multiple applications, ranging from photovoltaics and batteries to lasing and light-emitting diodes. The purpose of this Special Issue is to give an overview of the latest experimental findings concerning the tunability in composition, shape, functionalities, growth conditions, and synthesis procedures of perovskite structures and to identify the critical parameters for producing materials with functional characteristics.

Perovskite materials are currently receiving great scientific and technological attention because of their attractive properties and the easy deposition from solution for various application in diverse fields ranging from photovoltaics and batteries to lasing and LEDs. Their strong optical absorption, low non-radiative recombination rates, tunable band gaps, relatively high charge-carrier mobility, long diffusion lengths, and solution processability make them uniquely suited for these applications. This Special Issue comprises eight research articles and one timely review, and covers research in the field on new perovskite-based materials, improvements including electrodes and additives in solar cell active layers, and the importance of the atomic layer deposition process as a powerful tool for pinhole-free conformal thin films for similar applications. Furthermore, in-depth understanding of their photophysics in nanocrystals (NCs) films is provided. An interesting work is reported on the novel rapid photo-induced process to conjugate graphene-based materials with metal halide perovskite NCs [1]. A small number of laser pulses is sufficient to decorate the 2-dimensional (2D) flakes with metal halide NCs and tune the density of anchored NCs without affecting their primary morphology. This room temperature processing route provides unique opportunities for the design and development of perovskite–2D nanoconjugates which exhibit synergetic functionality for a wide range of applications.

In a different approach, in terms of fundamental research, a deep understanding of the photophysics in perovskite NC films is provided. It is important to understand the origins of the different behavior that are often observed in NC films with respect to bulk polycrystalline thin films or single crystals, and consequently to understand the origins of variations in the active properties to provide strategies for their improvement. The local morphological variations in a drop-casting thin CsPbBr_3_ NC film is exploited, showing the strong effect on the peak wavelengths of photoluminescence spectra, the linewidth, and the intensity of dependence on temperature from the aggregation of NCs [2].

Following the growth and characterization, the electrochemical performance of aerosol-assisted chemical vapor deposited phenethylammonium bismuth iodide in an aqueous solution of zinc sulfate as a proof of concept is evaluated estimating a specific capacity of 220 mAh g^−1^ at 0.4 A g^−1^ with excellent stability after 50 scans and capacity retention of almost 100% [3]. The high capacity value and the improved safety make the perovskite anode promising to utilize further in Zn^2+^ batteries.

An impressive research effort has also been directed in perovskite solar cells [4], reaching a record efficiency of 25.5% [5] and giving a place close to the best crystalline silicon performances. Zheng et al. reported the effects of 5-ammonium valeric acid iodide (5-AVAI or AVA) as an additive on methyl ammonium lead iodide perovskite solar cells [6]. A high stability is found due to both AVA and the protection by the all-inorganic scaffold achieving a power conversion efficiency of 14.4%. Parida et al. also reported the growth of transparent electrodes based on ITO nanoparticles by solution processing in ambient air without any heat treatment on Ag nanowires, exhibiting a power conversion efficiency of 5.64%, which outperforms the cell made with sputtered-ITO (4.14%) [7]. These findings open new pathways for the development of low-cost and quick-processing perovskite photovoltaics. Another interesting work is published by Meng et al. reporting the mesoporous titanium dioxide in perovskite solar cells [8]. The oxide is employed for the scaffold of the perovskite film and as a pathway for electron transport and the contact area between the perovskite and the titanium dioxide. The performance of the device based on the all-inorganic perovskite and titanium dioxide presented power conversion efficiency up to 6.39%. Finally, in the Special Issue, a simplified vertical structure of FTO–CsPbIBr_2_–carbon upon interfacial modification with polyethyleneimine species is suggested, yielding a power conversion efficiency of 4.9% with an open circuit voltage of 0.9 V and a fill factor of 50.4% [9].

Much effort has also been devoted to the development of organic solar cells, which can be fabricated through the low-temperature-based chemical process. It is found that the inverted non-fullerene bulk-heterojunction organic solar cells have better short circuit current density, leading to enhanced photovoltaic performance than the conventional solar cells [10].
